# An Unusual Case of Bilateral Hemorrhagic Chemosis in the Intensive Care Unit

**DOI:** 10.7759/cureus.9679

**Published:** 2020-08-12

**Authors:** Temitope Ajibawo, Erum Zahid, Yelena Leykind

**Affiliations:** 1 Internal Medicine, Brookdale University Hospital and Medical Center, Brooklyn, USA; 2 Pulmonary and Sleep Medicine, Brookdale University Hospital and Medical Center, Brooklyn, USA

**Keywords:** chemosis, intensive care unit, mechanical ventilation, lagophthalmos

## Abstract

Critically ill patients cannot complain about eye problems. Eyecare is often overlooked in the intensive care units (ICUs) because treatment is mainly focused on failures of organ which results in eye complications which are preventable. Therefore, we report a case of a patient admitted to the ICU who developed unusual bilateral hemorrhagic chemosis. Although, chemosis has been encountered often in the ICU, hemorrhagic chemosis without prior direct trauma is unusual.

## Introduction

Chemosis is defined as a collection of excess fluid within the conjunctiva tissue [1]. Factors predisposing to conjunctival edema vary and include increased ocular surface inflammation, venous congestion, and/or obstructed lymph drainage [2]. Chemosis can be caused by several conditions including infection, trauma, eye surgery, allergic reaction, and certain medications [1,3,4]. Chemosis is usually self-resolving or shows complete resolution after the elimination of the underlying condition [1]. In some patients, conjunctival edema may persist for more than four to six months, a condition known as chronic chemosis [5]. In this case report, our patient developed bilateral red-eye and swelling on day 10 of intensive care stay and was diagnosed with hemorrhagic chemosis. Herein, we present an unusual case of hemorrhagic chemosis that occurred in a patient admitted for status epilepticus in the intensive care unit (ICU).

## Case presentation

A 56-year-old African American female with a medical history of type II diabetes mellitus, asthma, chronic obstructive pulmonary disease (COPD), heart failure with reduced ejection fraction (HFrEF), hypertension, tobacco smoking and alcohol abuse presented to the ED with altered level of consciousness and new-onset generalized tonic-clonic seizures 3 hours prior to presentation. Upon the arrival of the emergency medical services (EMS), she was found on a chair at home having tonic-clonic seizures. There was no known history of head trauma, ocular or orbital trauma, and no prior history of seizures. 10mg injection of lorazepam and she was brought to the ED.

Upon presentation at the ED, initial vital signs showed blood pressure of 171/116 mmHg, pulse rate of 94 beats per minute, respiratory rate of 22 breaths per minute, temperature of 96.9^o^F and oxygen saturation of 93% on room air. Upon initial assessment, she was somnolent and unable to follow any commands. Examination of the eyes, lungs, heart, abdomen, skin and extremities did not show any abnormality. There were no obvious signs of injury. She was treated with a repeat dose of 10 mg IV lorazepam and loaded with 1g of levetiracetam. Due to persistent generalized tonic-clonic seizures and inability to protect the airway, she was intubated. The patient was admitted to the ICU. Initial laboratory values are shown in Table 1.

**Table 1:** Showing Initial laboratory values ALT, alanine Aminotransferase; AST, aspartate aminotransferase

Laboratory Test (Normal Range)	Initial
Hemoglobin (11.4-15.5 g/dL)	8.4 g/dL
White Blood Cells (4.5-10.2 x10 g/dL)	11.2 g/dL
Blood Urea Nitrogen (7-17 mg/dL)	40 mg/dL
Creatinine (0.52-1.04 mg/dL)	1.70 mg/dL
Bicarbonate (22-30 mEq/L)	9 mEq/L
Anion Gap (3-10 mEq/L)	27mEq/L
ALT/AST (9-5 U/L / 14-36 U/L)	686/667 U/L
Alkaline phosphatase (38-126 U/L)	561 U/L
Lactate (0.70-2.10 mmol/L)	9.20 mmol/L
Glucose (70-99 mg/dL)	845 mg/dL
Serum osmolality (280-296mOsm/kg)	345 mOsm/kg
Creatine kinase Total (30-135 U/L)	64 U/L
Alcohol Screen (0-10 mg/dL)	15 mg/dL
Magnesium (1.6-2.2 mg/dL)	1.3 mg/dL
Beta-hydroxybutyrate (0.02-0.27 mmol/L)	0.12 mmol/L

**Figure 1:**
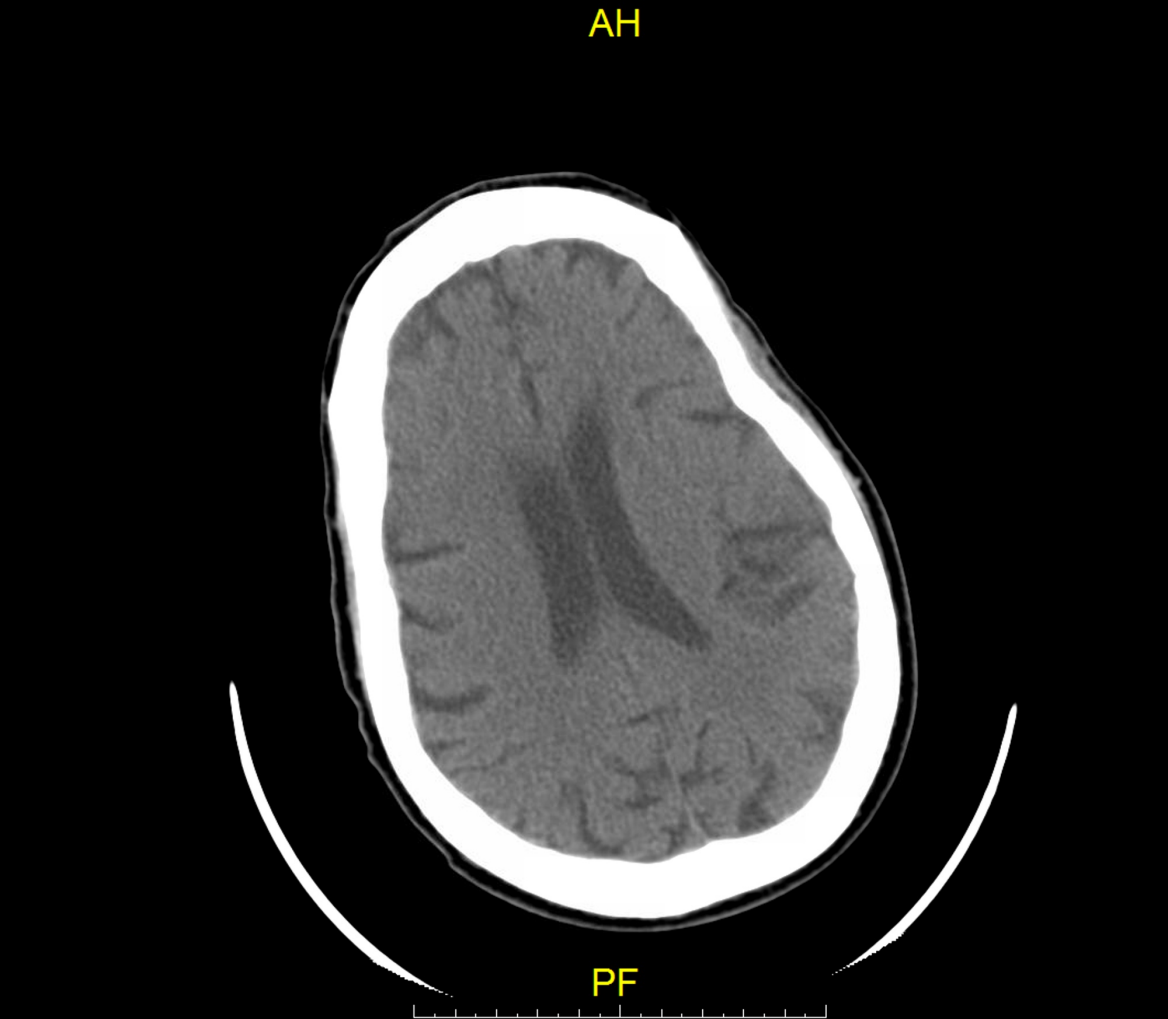
Initial CT head without contrast on arrival negative for acute infarct or hemorrhage

Initial CT head was negative for acute infarct, hemorrhage, masses, and/or bony abnormalities as shown in Figure 1.

**Figure 2:**
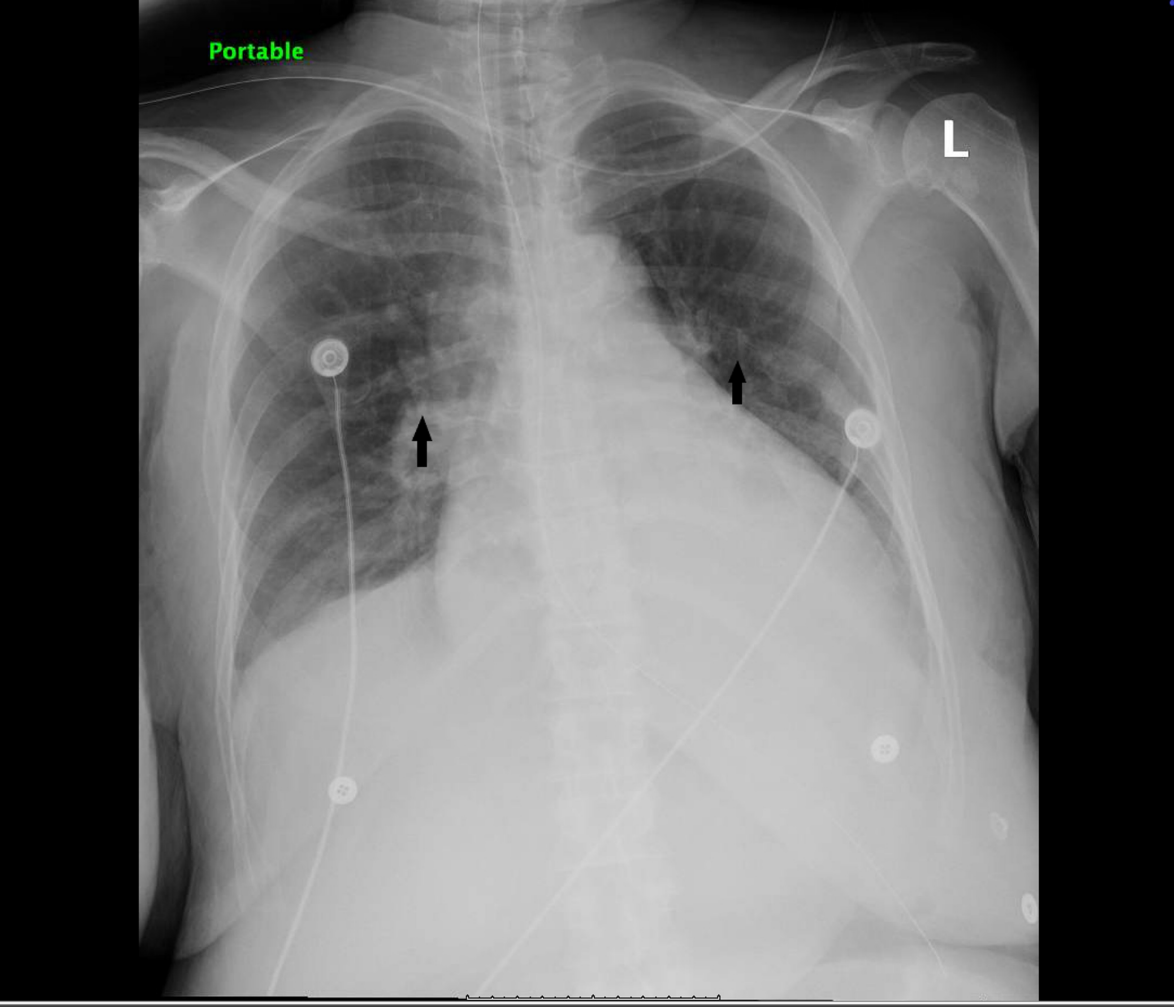
Chest X-ray showing infiltrates in bilateral lungs Infiltrates are indicated by the arrows

Initial chest radiograph showed bilateral infiltrates as shown in Figure 2.

Cultures were sent. Lumbar puncture was done and results were negative for meningitis or encephalitis. The hyperglycemic hyperosmolar syndrome was treated with isotonic saline and insulin infusion. She was initiated on broad-spectrum antibiotics (cefepime and vancomycin) which were stopped the next day after blood cultures came back negative. Given the patient's presentation during the COVID-19 epidemic, she was placed on airborne isolation and tested for COVID-19. Results of COVID-19 swab were negative. The patient was continued on levetiracetam, then lacosamide and valproic acid were added to control suspected subclinical seizures.

**Figure 3:**
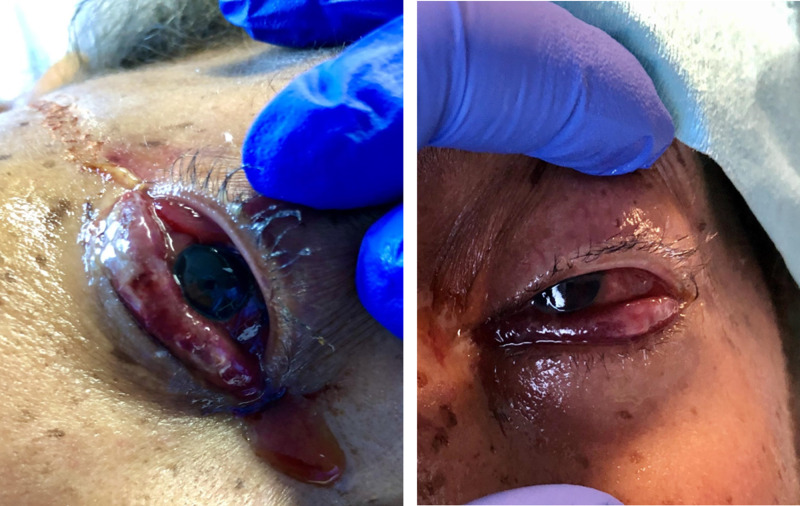
Image showing the right eye and the left eye with severe hemorrhagic chemosis

She remained intubated and on day ten post-admission, all the metabolic abnormalities had resolved but upon physical examination, the patient was found to have severe chemosis with bloody discharge as shown in Figure 3.

The patient was commenced on lubricant eye drops and ophthalmology evaluation was requested. Eye examination showed miotic, reactive pupils bilaterally, and blood-tinged chemosis overhanging lower lids bilaterally. The patient’s pupils were dilated with tropicamide and phenylephrine. Examination of the retina showed normal healthy optic nerves, along with flat and attached macular bilaterally. The ophthalmologist recommended starting 0.5% erythromycin eye drops one application to both eyes four times daily and maintain continuous eye lubricant drops. Conjunctival chemosis resolved completely after five days of treatment with erythromycin.

On day 17 post-admission, the patient was re-evaluated by the ophthalmologist, eye examination showed 2-3 mm round, reactive pupils bilaterally, no proptosis bilaterally, trace clear discharge bilaterally, no lagophthalmos, no chemosis and resolving diffuse subconjunctival hemorrhage bilaterally. An assessment of resolved hemorrhagic chemosis with resolving subconjunctival hemorrhage was made. Repeat CT head because of the persistent encephalopathy which did not reveal any change from prior CT head . The patient underwent electroencephalography (EEG) without sedation on day 19 post-admission, which showed generalized background slowing suggestive of moderate to severe encephalopathy but did not show any electrographic evidence of seizures. The patient remained encephalopathic, and she was scheduled for tracheostomy due to the inability to liberate her from mechanical ventilation. Severe acute respiratory syndrome coronavirus 2 (SARS-CoV-2) nasopharyngeal swab was repeated twice during admission and results came back negative.

## Discussion

We present an unusual case of bilateral hemorrhagic chemosis. The incidence of conjunctival chemosis in critically ill people ranges widely between 9% and 80% [6]. Chemosis is classified by the degree of conjunctival prolapse into mild, moderate, and severe chemosis. Mild chemosis is defined by the presence of slight protrusion of the conjunctiva; moderate chemosis, has more prominent conjunctival prolapse and severe chemosis means the presence of pronounced conjunctival prolapse that prevents eyelid closure [3].

The mechanism of chemosis leads to a continuous cycle of lymph channel disturbance and increased capillary permeability [7]. Chemosis course is believed to be generated by a positive feedback loop [4]. Conjunctival swelling causes extension of the fornix ligaments, which creates a potential space to contain serous fluid [7]. As the swelling increases, there is increasing conjunctival dehydration, prolapse of the eyelid, and interruption of tear flow over the surface of the eye [3,4]. Tear flow changes lead to an uncontrollable desiccation mechanism followed by Dellen formation [3]. Dellen formation is characterized by corneal epithelial loss and thinning eventually leading to worsening inflammation. The worsening inflammation re-initiates the positive feedback loop [3,4].

Possible contributory factors to conjunctival chemosis in our patient include prolonged use of positive pressure ventilation (PPV), and fluid & electrolytes abnormalities. Mechanical PPV results in increased jugular venous pressure, which disrupts venous return from ocular structures and eventually leading to fluid accumulation within visual structures [8]. In patients that are mechanically ventilated, ocular surface disorders tend to occur with positive end-expiratory pressure (PEEP) higher than 5cm H2O, which results in water and sodium retention and eventually conjunctival edema [9-11]. Our patients, like many critically ill patients, are prone to fluid and electrolytes imbalance, which makes them prone to conjunctival edema and impair eyelid closure [8]. Conditions such as hypoalbuminemia and fluid overload associated with generalized edema are risk factors for conjunctival chemosis [12]. In addition, states of increased hydrostatic pressure such as prone ventilation or prolonged recumbency predispose patients to conjunctival chemosis [12]. Conjunctival chemosis can also occur as a complication of eye surgery such as canthal surgery [3,4].

Critically ill patients are prone to loss of blinking reflexes, which can cause clinically significant eyelid edema by interfering with eyelid closure [12]. Sedated patients have impaired blinking reflexes, which result in significant loss of protective eye movements and lubrication [6,8].

Without a history of trauma, intraocular foreign body and eye surgery, our patient developed hemorrhagic chemosis which is unusual. Although there are predisposing factors for chemosis without hemorrhage in this case, the accompanying hemorrhage is unusual.

An ICU study by Suresh et al. used an eye care algorithm based on the assessment of eyelid position which showed 60% reduction in the incidence of chemosis [13]. Kam et al. also proposed an eye care protocol that emphasized clinicians should conduct an eyelid closure assessment (lagophthalmos) in a study of eye care in the critically ill [14]. The actions required are based on the grading of incomplete eyelid closure (grade 0-2) which include: grade 0 with no conjunctival exposures requires no intervention, grade 1 - some conjunctival exposure requires only lubrication and, grade 2 (some corneal exposure) - needs taping of the eyelids and lubrication of the eyes [14]. Early ophthalmology referral is crucial for prompt diagnosis, treatment and to prevent further complications [12].

## Conclusions

Our case highlights the risk of eye complications in intensive care patients if proper eye protocols are not followed. We recommend the implementation of measures which include regular ocular examination by ICU staff, taping of the eyelids if lagophthalmos is present and lubrication of the eyes in sedated patients which are not commonplace in ICUs. Early consultation of ophthalmologists is imperative for prompt diagnosis of ocular eye problems and prevention of eye complications.
